# Beyond literacy and numeracy in patient provider communication: Focus groups suggest roles for empowerment, provider attitude and language

**DOI:** 10.1186/1471-2458-9-354

**Published:** 2009-09-21

**Authors:** Doug Brugge, Timothy Edgar, Kelly George, Janette Heung, M Barton Laws

**Affiliations:** 1 Tufts University School of Medicine, 136 Harrison Ave., Boston, MA, USA; 2 Health Communication Program, 120 Boylston Street, Emerson College, Boston, MA, USA; 3 School of Communications and Theater, 2020 North 13th Street, Temple University, Philadelphia, PA, USA; 4 Harvard School of Public Health, 4/F Landmark Ctr., 401 Park Drive, Boston, MA, USA; 5 Tufts Medical Center, 800 Washington St, Boston, MA, USA

## Abstract

**Background:**

Although the number of people living in the United States with limited English proficiency (LEP) is substantial, the impact of language on patients' experience of provider-patient communication has been little explored.

**Methods:**

We conducted a series of 12 exploratory focus groups in English, Spanish and Cantonese to elicit discussion about patient-provider communication, particularly with respect to the concerns of the health literacy framework, i.e. ability to accurately understand, interpret and apply information given by providers. Within each language, 2 groups had high education and 2 had low education participants to partially account for literacy levels, which cannot be assessed consistently across three languages. Eighty-five (85) adults enrolled in the focus groups. The resulting video tapes were transcribed, translated and analyzed via content analysis.

**Results:**

We identified 5 themes: 1) language discordant communication; 2) language concordant communication; 3) empowerment; 4) providers' attitudes; 5) issues with the health care system. Despite efforts by facilitators to elicit responses related to cognitive understanding, issues of interpersonal process were more salient, and respondents did not readily separate issues of accurate understanding from their overall narratives of experience with health care and illness. Thematic codes often appeared to be associated with education level, language and/or culture.

**Conclusion:**

Our most salient finding was that for most of our participants there was no clear demarcation between literacy and numeracy, language interpretation, health communication, interpersonal relations with their provider and the rest of their experience with the health care system.

## Background

Many factors intrude on communication between patients and health care providers [1], including language, culture, education level, literacy and numeracy [2-4]. The roles of literacy, "the degree to which individuals are able to obtain, process, and understand basic health information" [5], and numeracy have been associated with health in multiple studies [6], and are reported to be important for navigating the health care system and making good health care decisions [7-9].

For people living in the United States with limited English proficiency (LEP), the impact of language on health communication has not been explored extensively [10-12]. Use of trained interpreters with LEP patients has been linked to reduced medical errors, greater patient satisfaction, improved health outcomes and compliance with medical regimens [13,14]. But the use of *ad hoc *interpreters such as family members, as well as poorly trained interpreters, was associated with miscommunication [15] and fabrication [16,17].

Prior experience translating public health surveys into Chinese and Spanish suggested to us that it was often difficult to effectively convey key health concepts, even after extensive back and forth between translators with considerable time at their disposal. This suggested to us that in the course of a clinical encounter communication with LEP patients would likely be compromised. Our starting hypothesis was that language would interact with education level and culture to influence accurate understanding of information given by health care providers, but these dimensions of health communication were not highly salient for our respondents. We chose to learn from our participants and adjust our thinking accordingly

We conducted exploratory focus groups in English, Spanish and Cantonese, languages that are distinct etymologically [18]. We viewed this study as formative research designed primarily to generate ideas for future research.

## Methods

### Recruitment

Participants were recruited in and near Boston, Massachusetts by "recruiters" who were knowledgeable about specific target populations (for example, low income Chinese immigrants). They recruited through the recruiters' social networks by word of mouth in a snowball type approach. We did not keep records of refusals, but recruiters reported that scheduling was the main reason for not participating.

For Spanish speaking participants an inclusion criterion was reported Puerto Rican or Dominican origin, because these are the predominant Spanish-speaking populations in Massachusetts, and they have considerable cultural affinity. As Chinese speakers generally do not understand multiple dialects, our inclusion criterion was speaking Cantonese, the majority dialect in Boston Chinatown. To narrow the cultural range of English speaking participants, we required that they report being white non-Hispanic. We sought a mix of men and women over the age of 18.

We recruited low and high education level participants as a surrogate for literacy level because there is no literacy screen validated across three languages. Low education level was defined as no more than a high school education. High education level was defined as 2 years of college or more.

Following standard practice, we excluded anyone who worked in advertising, research, public relations or promotions, the media, or health care. We also sought to exclude people who (semi)professionally did written "translation" but failed to ask about oral "interpretation". Consequently there was one high education Spanish language participant who had done paid medical interpretation. We also excluded members of the clergy, feeling that they might inhibit or dominate a discussion. We did not restrict groups to people who did not previously know one another and, in several cases, participants were clearly acquainted with one another prior to the focus group. Participants were paid $50 and given a light meal.

### Translation of consent and guide

The consent forms, screening form and facilitation guide were translated into Spanish and Cantonese by translators with strong verbal and written capacity in both languages and years of professional experience. They were back translated by equally qualified bilingual translators who had not seen the original English. The final versions were produced by reconciliation between the translators.

### Locations

We used focus group suites which had observation rooms with one-way mirrors at either Tufts University School of Medicine or Emerson College. We videotaped the groups via cameras that allowed clear identification of each speaker. Sound was recorded through high-sensitivity microphones. One of us observed all of the focus groups accompanied by interpreters who summarized 6 of 8 non-English sessions.

### Focus group methodology

We conducted 12 focus groups that ran for approximately two hours each (including the consent process) from August 2006 to February 2007, 4 in each language, stratified into 2 low and 2 high education sessions. Low education was defined as no more than a high school education while high education was 2 years of college or more. We reasoned that literacy would be lower on average for the low-education participants, as there is no literacy assessment validated across three languages.

The Spanish and Cantonese sessions were led by individuals who were fully bilingual and were ethnically concordant with participants. These facilitators had prior experience leading research focus groups in their respective languages and met with the lead author to prepare. The English focus groups were facilitated by one of us, also ethnically concordant, who has extensive experience conducting focus groups. The facilitators were not involved in recruitment, transcription or translation.

To standardize the focus groups across facilitators and sessions, two of the authors (DB, TE), one of whose professional expertise is in conducting focus groups (TE), developed a detailed guide. Facilitators were instructed to maintain a neutral, but cordial approach and generally adhered closely to the guide, but had flexibility to probe discussions and lines of thought as they arose. The guide had three sections:

1) Participants were asked to discuss negative and positive experiences that they had when communicating with their health care providers. The guide read, "...try to think of examples of visits you have had where you thought the provider did a good job of communicating/speaking and also ones where you thought the provider did a poor job of communicating/speaking...examples can refer to a diagnosis or to instructions for using medication or something else."

2) Participants were then asked to define toothache and depression. Our goal was to gauge the range of variation among the language groups in cognitive understanding of these terms. We thought that toothache was so concrete that understanding of the word would vary little across languages. We suspected that depression was a culturally laden term that could be understood in various ways, from sadness to a physiological illness.

3) Respondents were also presented with a set of dialogues between doctors and patients that did not result in substantial additional qualitative data. They did, however, provoke personal stories from two Latina respondents, that we coded those as part of the overall responses to the communication issue.

We conducted a detailed review of facilitation after transcription and translation. Facilitation of the Cantonese and English focus groups was excellent in terms of following the written guide, remaining neutral and probing for follow-up. Facilitation of the Spanish focus groups included some digression and failure to probe adequately, but only a few instances in which the facilitation was leading, which we excluded from analysis.

### Translation and transcription

English language focus groups were transcribed by one person and checked against the video by one of the authors (DB), showing high accuracy. Cantonese focus groups were translated and transcribed in a single step by one bilingual Cantonese-English speaker/writer. The transcripts were checked for quality by comparison to the video by a second, equally qualified translator (author JH) and resolved by consultation between the translators. The Spanish focus group videos were transcribed by native Spanish speakers. The transcription was spot checked by one of the authors (MBL) as it proceeded and questions resolved. Transcripts were translated from written Spanish into English by a bilingual writer (author BL) in consultation with Puerto Rican native speakers.

### Analytic approach to data analysis

We conducted a systematic content analysis to label and categorize the data. Resulting categories were examined through thematic analysis. Themes could be articulated directly by the participants or identified by the study team.

We began by having three of the authors (DB, JH, MBL) analyze a single low education Cantonese transcript. They compared their analyses and agreed upon a preliminary coding system. Subsequently, one of them (JH) coded the remaining Cantonese transcripts and her analysis was reviewed by one of the authors. Two authors (DB, MBL and DB, KG) coded single English and Spanish focus groups and met again to reconcile their coding, finding that it differed little in content, but only in organization. Authors MBL and KG then coded the remaining focus groups and their coding was reviewed by a fourth author (TE). In the Spanish focus groups the facilitator often failed to confirm whether language discordance existed in cases where this seemed likely based on context. We coded these instances as discordant for language.

Our final step was a logical analysis looking for patterns of difference, as well as similarities that emerged from cross-classifying the data. Quantitative counts of themes were put into broad categories (Table 1) to diminish the temptation to conclude that small quantitative differences were meaningful. We chose quotes that were broadly representative, relatively articulate and, in more limited instances, that illustrated countervailing views.

**Table 1:** Demographics of participants in each focus group

	**N**	**%**	**%**	**%**	**%**	**%**
	
		**Male**	**White**	**Asian**	**Latino**	**Low education**
English Low Ed. 1	4	0	100	0	0	100

English Low Ed. 2	6	67	100	0	0	100

English High Ed. 1	8	50	100	0	0	0

English High Ed. 2	5	20	100	0	0	0

Cantonese Low Ed. 1	7	14	0	100	0	100

Cantonese Low Ed. 2	9	56	0	100	0	100

Cantonese High Ed. 1	4	25	0	100	0	0

Cantonese High Ed. 2	10	60	0	100	0	0

Spanish Low Ed. 1	12	8	0	0	100	100

Spanish Low Ed. 2	5	60	0	0	100	100

Spanish High Ed. 1	5	100	0	0	100	0

Spanish High Ed. 2	10	50	0	0	100	0

### Research Ethics

The study was approved by the Tufts-New England Medical Center IRB and is in compliance with the Helsinki Declaration. All persons employed by the study completed mandated human subjects training. Participants were assured of confidentiality by controlling access to original recordings and removing identifiers during transcription. The research team reports that they did not occupy duel roles (e.g., clinician and researcher).

## Results

### Study Sample

A total of 85 people participated in the 12 focus groups, a number limited by our resources. Focus group size ranged from 4 to 11 participants (Table 1). All participants in the Cantonese groups identified as being Chinese and all participants in the English groups identified as being white. All Spanish language participants were of Latin American origin. However, two of the high education Spanish participants identified as "other" rather than Puerto Rican or Dominican. One appeared to be from Mexico based on comments made during the session.

Fifty-seven percent of the participants were women, with some focus groups unbalanced by sex (Table 1). Based on observation, we had a good range of ages overall, while some groups had a narrow age range. We did not collect quantitative information on English fluency, but based on observation nearly all of the high education participants in Cantonese and Spanish could communicate in English, while almost none of these low education participants spoke English well.

### Thematic Analysis - Positive and negative experiences

As this was an exploratory study, we analyzed themes that were not explicitly related to accurate understanding because they arose frequently as salient concerns of participants. The themes we identified were:

1) *Language discordant communication *included use of medical interpreters, and communication in which the provider and patient shared little or no common language;

2) *Language concordant communication *encompassed health communication in which the provider and patient shared the same language. This included issues related to literacy and numeracy, such as use of technical jargon;

3) *Empowerment/disempowerment *could, for example, involve a patient speaking up (or not) to their provider, or taking the initiative to research health information;

4) *Providers' attitudes *addressed how the patient perceived being treated, for example, the provider being rude or friendly;

5) *Issues with the health care system (all coded as negative) *that were not primarily defined as provider-patient communication, such as rushed visits or difficulty scheduling an appointment.

### Quantitative distribution of themes

Table 2 provides the quantitative occurrence of themes stratified across the 12 focus groups.

**Table 2:** Themes by language and education level

	**Language discordant communication**	**Language discordant communication**	**Language concordant communication**	**Language concordant communication**	**Provider attitude**	**Provider attitude**	**Empowerment**	**Empowerment**	**Health care system**
	**Positive**	**Negative**	**Positive**	**Negative**	**Positive**	**Negative**	**Positive**	**Negative**	

**English Low** **Ed. 1**	None	None	Occasional	**Common**	Occasional	**Common**	Occasional	Occasional	Occasional

**English Low** **Ed. 2**	None	None	Uncommon	Uncommon	Occasional	Occasional	**Common**	**Common**	**Common**

**English High** **Ed. 1**	None	None	**Common**	Uncommon	Occasional	Occasional	**Common**	Uncommon	Occasional

**English High** **Ed. 2**	None	None	Occasional	Uncommon	**Common**	Occasional	**Very common**	Uncommon	Occasional

**Cantonese** **Low Ed. 1**	Occasional	Occasional	**Common**	Uncommon	Occasional	Occasional	Occasional	Occasional	None

**Cantonese** **Low Ed. 2**	Uncommon	Uncommon	Occasional	Occasional	Occasional	**Common**	Uncommon	**Common**	None

**Cantonese** **High Ed. 1**	None	None	Occasional	None	Occasional	Occasional	**Very common**	None	None

**Cantonese** **High Ed. 2**	Uncommon	Uncommon	Uncommon	None	**Common**	**Common**	**Common**	None	None

**Spanish** **Low Ed. 1**	Uncommon	Occasional	Occasional	Uncommon	Occasional	Uncommon	Occasional	Occasional	Uncommon

**Spanish** **Low Ed. 2**	Uncommon	Occasional	Occasional	Uncommon	Uncommon	Uncommon	Uncommon	Occasional	Uncommon

**Spanish** **High Ed. 1**	None	None	Uncommon	Uncommon	Uncommon	Uncommon	Uncommon	Uncommon	Uncommon

**Spanish** **High Ed. 2**	None	Uncommon	Uncommon	Uncommon	Uncommon	Uncommon	Occasional	Occasional	Uncommon

Main themes raised during the positive and negative experiences discussions, categorized as none (0), uncommon (1-3), occasional (4-9), common (10-19; **in bold**) and very common (20 or more; **in bold**).

#### English

There were no reports of language discordant communication in the English language focus groups. Language concordant communication did come up, but it was a small part of the conversation, often prompted by efforts of the facilitator to steer discussion to the issue of accurate understanding. Stratified on education level, participants in the low education English focus groups reported more negative experiences and less positive experiences regarding empowerment issues and language concordant communication than did the high education English participants. There was also a trend in this direction for provider attitude with the raw counts, but less clearly when put into the categories in the table. All of the English language groups brought up issues of the health care system in spite of attempts to steer the conversation to communication.

#### Cantonese

Language discordant communication was a more substantial point of discussion in one of the low education Cantonese groups than in the other. The issue of interpreters also came up in one of the high education groups. Both positive and negative views of interpretation emerged. The lower educated groups tended to report more negative empowerment experiences while the higher educated reported more positive experiences. The high education groups mostly discussed language concordant communication with Cantonese-speaking providers. There was no obvious trend with regard to education level in terms of positive and negative comments about provider attitude. Themes related to the health care system were not raised.

#### Spanish

Language discordant communication, mostly negative, was discussed in both low education Spanish focus groups, but came up in only one of the high education Spanish sessions. While empowerment and provider attitude were discussed in all the Spanish focus groups, there was not a trend toward negative or positive comments, nor was there much difference across education levels. Discussion of language concordant communication issues tended to be positive, although negative examples arose. Low education groups raised more positive examples of concordant communication than did high education groups. Health care system concerns were raised in all the Spanish groups, but less so than in the English sessions.

### Qualitative assessment of English groups

#### Health communication

The English language participants did not want to talk about instances of misunderstanding in health care situations and had to be prompted repeatedly to address the issue. When they did speak about understanding, low education participants spoke of the need to "cut out the jargon" and pointed, when prompted, to print materials that helped them adhere to behavioral change. For the higher education participants, examples were more sophisticated, including "drawing me a molecular diagram."

High education English groups were particularly interested in getting credit for their knowledge and intelligence. Many of their comments were related to negotiating with providers for power, time, and especially more information. These participants emphasized the personal research that they conducted (Appendix 1, Quote 1).

#### Personal relationships

The low education English groups were more interested in personal relationships. Many of their comments concerned how the providers made them feel through eye contact, empathy, listening or being friendly versus being businesslike. They wanted providers to speak like laypeople (Appendix 1, Quote 2). Despite some positive experiences, examples were frequently disempowering for low education participants (Appendix 1, Quote 3).

#### (Dis)empowerment

Low education English expressions of empowerment were basic, "...I think you need to be better prepared when you are interfacing with your physician....I feel as though you should provide your physician with all the medication you are taking. All your symptoms. And even if you have to write it before-hand..." These groups also related many negative examples of provider attitudes, " [The provider] was kind of rude and rushed....That day, the moral of the story is that he...rushed me. And it was only a few minutes, but he coulda taken an extra 5 minutes, but the way he was kinda, really, yelling."

High education participants tended to be more positive, ""I mean that's the relationship that I have with him. He always gives me a ton of information because he knows if he doesn't, I'm going to ask him ten thousand questions. He's being proactive."

### Qualitative assessment of Cantonese groups

#### Language

Language discordance was discussed in both the high and low education Cantonese focus groups, but did not dominate. Both positive and negative examples arose. A bilingual highly educated participant noted a preference for Chinese, "You feel different when you are meeting with a Chinese doctor. I understand English, but Chinese is easier to get into my mind. You have different feelings with it." One participant in a low education group indicated that it was helpful that an Anglo doctor knew some words of Mandarin, even though that was not the participant's first language.

There were numerous examples of language interpretation that participants considered inadequate. One said, "... the problem is that the interpreter only said a few sentences when the doctor said a lot. That's why I wasn't satisfied. The doctors were very good." A low education participant summed it up, "I did not understand what the doctor said; I just believe whatever the interpreter said. The big problem is we do not understand English."

#### (Dis)empowerment

As with the English groups, the high education Cantonese speakers were more likely to give examples of empowerment (Appendix 2, Quote 4). A low education participant, in contrast, reported making a suggestion to their health care provider regarding something they disagreed about, but did not report researching the issue.

The high education Cantonese speakers reported no examples of disempowerment, but some low education Cantonese participants had strong opinions about their position of inferiority. One said, "I was afraid to ask. They are the doctors and we are the patients. I don't want to seem like I was nagging them, and I was afraid to annoy them." Another noted, "They thought we didn't have the education and so we know nothing."

#### Provider attitude

Provider attitude, both positive and negative was of significant interest to Cantonese speaking participants at both education levels. Said one low education participant, "Encourage me, hug me. No racist feeling. We were in different cultures, spoke different languages, but they used some gestures [or body contact] that made me feel close to them, and that resolved the nervous mood."

A high education participant said, "One thing I really liked about the doctor was not just his good language skills - it was how he used books and pictures as illustrations of the disease. In fact, besides pictures, he even used a human model to explain the etiology of the cancer and its location."

These examples were countered by some negative ones. A low education Cantonese speaker expressed frustration, "He just checked this [boxes on a form] and checked that. Then I could leave. That is it. Nothing is wrong. That kind of attitude, I feel it is sometimes very flippant [dismissive]." A high education focus group member observed that, "For other doctors, they were not 'gentle' enough. So when they approached patients, it was not as easy for patients to accept them; the patients didn't trust them as easily."

### Qualitative assessment of Spanish groups

#### Language

Spanish speaking participants didn't readily respond with reference to instrumental communication as such. Rather, they tended to describe experiences with illness and health care in which communication was not clearly separated from other elements. The low education Spanish language participants in particular had recurring complaints about the skills of hospital interpreters. Some participants preferred to have their daughters or granddaughters interpret. One specific observation was that interpreters born in the U.S. of Spanish-speaking parents only think that they speak Spanish well (Appendix 3, Quote 5).

#### Complex stories

In the Spanish groups participants often related complex stories that we coded for multiple themes. Failure to effectively convey important health information came up repeatedly in these focus groups, but participants' ability to understand information accurately was not generally central (Appendix 3, Quote 6).

A participant in a low education group reported, "I have a problem with the lungs, so then I have to see a lung specialist, an American, they don't speak Spanish. In other words, I quit them." But not because of the language barrier, as she had an interpreter who apparently did a satisfactory job, rather the issue was that the pulmonologist insisted that she did not have asthma, when, she maintains, she did. She says she became gravely ill, and end up changing to a Mexican physician.

#### Culture

Like the Cantonese speakers, many Spanish language respondents reported preferring culturally concordant physicians. The recurrent theme is that Latino physicians have more warmth, that Anglos are too cold and businesslike. Nevertheless a participant in one of the high education groups decided that technical skills were more important (Appendix 3, Quote 7). Another participant in the same group was less tolerant of the Anglo attitude. With his Cuban physician "I feel that I am speaking with a person, not a robot... the Anglo Saxons don't like to radiate that human warmth."

#### Other factors

Age, sophistication and personal connections, not just ethnicity and English ability, were perceived by some participants as affecting how people were treated. In one case, a story revolved around a male participant seeing a female doctor and being uncomfortable with the digital rectal exam. In another instance, because of the distress that a participant was feeling at the time, she failed to mention her allergy to her physician and took the pill without bothering to find out if it was correct for her or not. She appeared to blame herself.

### Thematic analysis - Key words

#### Toothache

There was little distinction between high and low education focus groups in English or Cantonese with respect to defining toothache. Across the English groups the main focus was on pain in the tooth or mouth. Some of the descriptions were creative such as a bee sting in the mouth, but the underlying meaning seemed consistent across groups. The Cantonese sessions also focused on pain, while referencing inflammation, gingivitis, and X-rays. There was more distinction between the low and high education Spanish groups. The high education participants gave dictionary definitions and etiological theories, were more abstract, unemotional, and detached while low education Spanish groups often provided emotionally laden personal narratives.

#### Depression

The low education English participants mostly described what it feels like to be depressed, including not wanting to get out of bed, being sad or tired all the time, and being hopeless. Suicide was seen as a risk. Descriptions from the high education English-speaking participants were more complex and colorful. They included words like "morosity," despondent mood, psychological and emotional state, irritability and the myth of Oroborus, the snake that ate itself. One of the high education groups also touched on issues of biochemistry and nature vs. nurture.

Two low education and one high education Cantonese focus group discussed depression in terms that were similarly descriptive (how it feels) and that attributed depression to social/environmental causes, including bottling things up inside and adverse life events. The word used by the facilitator for depression could be translated literally as, "suppressed melancholy" (). Subsequently we determined that there are other synonyms, such as "worrisome melancholy" (). It could be that the word choice influenced the discussions. In contrast, the remaining high education focus group discussed the roles of "chemicals," hormones, psychology and medications.

Similarly, the high education Spanish groups included discussion of biological and social causes, referencing neurotransmitters and the impact of traumatic events, while the low education groups offered personal experience, including crying a lot and not wanting to eat or do anything.

## Discussion

### Main point

We began this study with the assumption that issues of literacy and numeracy, well-studied core aspects of health communication and, in addition, language would emerge as predominant themes and that, perhaps, these themes would be distributed unevenly by language and education level. Although these themes were present in most of the focus groups and there was appreciable variation by language and education level, the participants were not satisfied staying within the boundaries that we tried to draw for them. They insisted on or slipped into talking about other related aspects of health care, sometimes despite repeated prodding by facilitators to stay on topic. Participants wanted to talk about provider attitudes and how they felt empowered or disempowered during interactions with their providers. The English language participants and, to a lesser extent the Spanish language participants, also wanted to talk about the health care system more broadly.

We interpret this as evidence that the conventional focus on literacy and numeracy, while important, is not the most salient frame for most patients. Certainly, for the low education, LEP participants it seems clear, and not surprising, that language is an important barrier to effective communication. But beyond these basic factors -- literacy, numeracy and language -- are elements of social relationships, coded as (dis)empowerment and provider attitude themes in our analysis, that were clearly very important to our respondents.

For most of our participants there was no clear demarcation between health literacy, health communication, interpersonal relations with their provider and the rest of their experience with the health care system. Participants seldom volunteered examples of difficulty understanding what a provider told them; rather, they were likely to complain about not being told anything at all. We think that our findings fit within frameworks that have been put forward in previous scholarship. We note that our participants seem very much to be grappling with issues of power asymmetry and voices of the "life world" and "medicine" as discussed by Mishler (1984) and Gwyn (2002) [19,20]. Similarly, Ong et al. (1995) in their review of patient-doctor communication, distinguish "creating a good interpersonal relationship" and "medical decision-making" in addition to "the exchange of information" [21]. Further, they point to the emphasis in the literature on instrumental-focused exchange at the expense of the role of affective behavior and point to socio-demographic factors that might modify patient-doctor interactions.

We think that the frameworks that these authors propose have significant explanatory power for our data. One specific point is that our analysis tends to support the role of socioeconomic status cited by Ong et al. (1995) [21]. Our low education participants showed a greater concern with the interpersonal aspects of their encounters with health care providers, although themes of this nature also appeared to a lesser extent in the higher education groups.

The concept of "patient-centered" health care is widely accepted in medicine, and one might even conclude that, especially for most of the higher education participants, that what they seek is patient centeredness. However, as thoughtfully developed arguments of de Haes (2006) point out, patients do not necessarily want unlimited information from their providers, nor do they necessarily want a role in medical decision-making [22]. Although we did not explore this point explicitly, it is interesting that our low education participants seemed far less concerned about seizing control of their medical fate than did our high education participants.

### Secondary points

1) The health communication literature is thin in terms of the independent role of language relative to literacy and numeracy. Indeed, we are aware of no validated measure that takes into account English proficiency when assessing health literacy in LEP populations. A very recent quantitative study [23] reported independent roles for language and health literacy in patient provider communication. Our focus groups tended to confirm important roles for education level (a surrogate for literacy and numeracy in our study) and language as meaningful factors that affected communication between these patients and their health care providers. This would seem to point to the need for developing a framework for understanding, assessing and addressing health literacy for LEP people.

This research will need to consider the context of health care for the patient. That is, if they are they receiving care from a provider that speaks their language (in which case literacy in their native language should be sufficient), the literature suggest that this will partially mitigate the language issue [24-26]. They might also be receiving care through an interpreter (of whatever quality) or be dependent on whatever English skills they have. While there is an indication that interpretation affects quality of medical encounters [27], there is much more work to be done on how to disentangle medical interpretation from the technical barriers that exist even in English, and the cultural and power relationships. Medical interpretation was highly relevant for the lower educated Cantonese and Spanish speakers. These non-English-speaking participants had to grapple with interpreters that they often perceived as providing inadequate interpretation for many reasons. As stated simply by one Chinese participant, "The big problem is we do not understand English."

2) The role of education was particularly evident in relation to the empowerment theme, with highly educated participants only rarely sharing experiences of disempowerment that were relatively common among the less educated participants. This pattern was particularly apparent in the English and Chinese focus groups. We suspect that what we captured reflects a basic social aspect of interpersonal relations between patients and providers. By assessing communication across education levels rather than by literacy level, it is possible that we saw attitudes of health care providers that differ by social status of the patient. It is also possible that this is mostly a perception of the participant arising from their own feelings of inferior social status. Alternatively, it is possible that literacy and numeracy are driving this effect indirectly. However, for that to be the case, one would need to explain why the participants were uninterested in talking about the instrumental effectiveness of communication and even, in many instances, resisted doing so.

3) We also noted a desire for cultural congruence by many, but not all, Cantonese and Spanish language participants. This was most obvious in the high education participants who spoke English, but still had a preference for Chinese or Hispanic health care providers. This is consistent with other reports that Asian and Latino patients are more likely to report feeling disrespected and less positive interactions with their physicians [28,29].

4) Another observation was that the high education respondents were much more likely to offer abstract analysis and generalized judgments about physicians and the health care system, particularly noteworthy in the Spanish high education focus groups. The low education groups featured less of this, and stuck to narratives about specific personal experiences, although such narratives certainly appear in the higher education groups as well. This difference is particularly notable in the responses to defining depression, where the higher education participants often provided biomedical definitions and etiological theories, whereas the lower education participants responded mostly in terms of personal experience.

### Strengths and limitations

A substantial strength of this study is that, as far as we are aware, this is the first investigation of health communication across three languages and two education levels. Therefore we could begin to explore the roles of language and culture that have been less well studied than basic literacy and numeracy. Another strength that emerged was our exploratory framework, such that our focus groups were not narrowly designed and this allowed participants to push back against our expectations. This, in turn, forced us to think about issues beyond literacy, numeracy and language.

Working in three languages presented methodological challenges, including logistics and standardization across languages using different facilitators. While the English and Cantonese focus groups were facilitated very similarly; the Spanish focus groups were not as consistent, which might partially explain the difference in patterns of themes in these groups relative to the other languages.

A significant structural limitation of this study is that we heard only from patients, and not from providers and interpreters. These parties could report substantially different versions of events than do patients. Patients are likely inclined to press their own grievances and downplay their shortcomings in health care interactions.

We had only two focus groups for each demographic category and recruited participants from a single metropolitan area. Our findings cannot be assumed to be generalizable, both because the participants are not a representative sample and because the number of participants is small.

## Conclusion

Our study is only a first step toward exploring health communication in LEP patients in the US. Given the limited literature in this area, it is too early to draw any firm conclusions; however, we do think that our findings suggest future research directions. There is a need for additional focus group studies with larger numbers of participants that include health care providers and medical interpreters. These studies should be designed to explore issues beyond literacy and numeracy. There are also two complimentary areas of work required. One is cross-sectional surveys that can gauge opinion of representative samples of patients, providers and interpreters. The other is direct recording of patient-provider visits in order to get past the bias of recall and distortion of self-interest. Methodological tools for rigorous analysis of cross-language communication are needed.

## Appendix 1

### English quotes

*Quote 1: *(English High Ed) I Google [referring to the Internet search engine] my eyes off about bad stuff and I try to be as informed about health issues, drug interactions, contraindications, eastern, western, conventional, unconventional as I can.

*Quote 2: *(English Low Ed) But the way they explained everything, and the way they treated me, like a human being! Like a person. Just from being there with them, I felt, I felt so much better. I was in real pain... but it felt just good to be there. And, know I was in safe hands, and know that they were helping me.

*Quote 3: *(English Low Ed) but you know, just that idea that we're somehow regular folks, and that the doctors and the professionals are somehow you know, over here [pointing upward]....it really stems from that idea that societally we're just separated. And there's just sort of this moat. In the moat, maybe there's money in the moat. Or, degrees or whatever. But that seems to affect people on both sides of that moat.

## Appendix 2

### Cantonese quote

*Quote 4: *(Cantonese High Ed) If the doctor used medical terms that I didn't understand, then like [participant 2], I would ask the doctor to explain a little more. And even after his explanations, after I got home, I usually would still research more on the topic, for example using the internet or the dictionary.

## Appendix 3

### Spanish quotes

*Quote 5: *(Spanish Low Ed) One participant: Sometimes ... it seems that they [interpreters] don't understand well, what one says, and they interpret it in their way, even though all the Hispanics, we aren't of the same country.

Second participant: Some words are different.

First participant: Some words they don't, don't understand them.

Third participant: ...they are Hispanic because dad and mom are Hispanics, not because...they were born here.... there are many [interpreters]...born here and they don't know many words [in Spanish].

Fourth participant: Frequently the physicians, because they are so well educated, know Spanish, but the problem is that sometimes they use the medical language and sometimes the interpreter doesn't even understand it...

*Quote 6: *(Spanish Low Ed): [Participant is having a liver biopsy.] They didn't give me any kind of explanation. They left me thrown there, they merely said to me, "Let's go to such and such a room," right then, and they didn't explain anything. So I am looking this way but, "Where am I going?" So it was that another person [finally?] came...when I was about to fall asleep [apparently from anesthesia]...He/she explained to me...Now is when you're going to tell me this?

Facilitator: But can you ask?"

Participant: Yes I can ask ... that is true, it is my negligence because it is I who is going to undergo treatment. But, uhhh, it didn't occur to me. I thought that they were going to [explain] it to me.

*Quote 7: *[Spanish High Ed] He [a referring physician] gave me two alternatives: one type who was Ecuadorian and apparently of this great loveliness (*aparentemente es Latino de estos bien buenos*) and another type who is a gringo which is a cold type.... So I say to him, which of them do you believe is better. He tells me, I believe that the gringo is better... Give me the gringo, no?

## List of Abbreviations

LEP: Limited English Proficient

## Competing interests

The authors declare that they have no competing interests.

## Authors' contributions

DB designed and directed the study, including data collection and analysis and took the lead on writing the manuscript. BL participated in the analysis and writing of the manuscript. KG participated in the data collection and analysis and reviewed and approved the manuscript. JH participated in the data collection and analysis and reviewed and approved the manuscript. TE helped design the study, participated in the data collection and analysis and contributed to writing the manuscript. All authors read and approved the final manuscript.

## Pre-publication history

The pre-publication history for this paper can be accessed here:


